# Supplementary material for: Are carbon emissions trading and green financial instruments synergistic? -Comprehensive quantitative research based on content analysis
Source: PLoS One. 2024 Mar 7;19(3):e0298601. doi: 10.1371/journal.pone.0298601 (PMC10919601; doi:10.1371/journal.pone.0298601)
Supplement: S1 Data — (ZIP) [file pone.0298601.s001.zip › Data/地级市Z_F工作报告.zip!/╣π╬≈/▒▒║ú╩╨/└┤╫╘╣1⁄2╓┌║┼í╛┬φ┐╦╩2╛▌═°í┐_2019.docx]

各位代表：

现在，我代表市人民政府，向大会报告政府工作，请予审议，并请市政协委员和列席会议的同志提出意见。

一、2018年工作回顾

过去一年，在自治区党委、政府的正确领导下，我们以习近平新时代中国特色社会主义思想为指导，深入学习贯彻党的十九大精神，全面落实习近平总书记视察广西及北海重要讲话精神，认真贯彻落实市委的工作部署，在市人大、市政协的监督支持下，坚持稳中求进工作总基调，积极应对经济环境新变化，主动适应区域合作与竞争新形势，提高站位、拓宽视野，跳出北海看北海，对标东部沿海开放城市，立足当前、放眼未来，着力提高城市综合竞争力，实施差异化发展战略，完善北海新一轮发展的谋篇布局，找准突破口和着力点，创新一揽子发展机制和工作举措，加快打造向海经济，突出抓好招商引资和项目建设，在宏观经济下行压力增大的背景下，全市呈现出逆势向上的发展态势，开启了赶超跨越新征程！

2018年，全市地区生产总值增长8.3%，规模以上工业总产值增长14.5%，规模以上工业增加值增长7.1%，固定资产投资增长8%，财政收入增长12.2%、达225.2亿元,增幅排全区第二位、总量排全区第四位，税收占财政收入的89.6%，财政收入质量居全区之首，经济社会继续保持健康快速发展。

一年来，我们主要做了以下工作：

（一）抓好主导产业和园区经济，工业发展迈出新步伐

以产业园区为载体，以主导产业为重点，强龙头、补链条、聚集群，工业产值保持两位数增长，规模以上工业企业税收达到132亿元，占全市税收的65.4％，对经济发展的支撑作用持续增强。

主导产业日益壮大。电子信息产业发展势头强劲，一批电子信息产业项目落地建设，惠科移动智能终端一期项目投产，中电北部湾信息港入驻企业186家，北部湾表面处理中心基本建成，惠科电子北海产业新城顺利推进。石化产业稳步发展，国内最大的催化汽油吸附脱硫装置建成投产，中航化项目竣工，北海炼化结构调整项目获得批准。临港新材料产业加快聚集，诚德公司“填平补齐优化”项目新增两条固溶生产线。总投资超百亿元的信义玻璃产业园开工建设，成为临港新材料产业未来新的增长点。

园区经济后劲增强。园区基础设施进一步完善，一批项目、企业入驻，聚集能力不断增强，成为工业发展的主战场，五大园区工业产值占全市规模以上工业总产值的92.3%。北海工业园区企业突破1000家，铁山港（临海）工业区成为千亿元产值园区、实现税收123.6亿元，北海综合保税区通过国家验收，北海高新技术开发区在全国综合排名从88位提升至79位，北海海洋产业科技园区引进20余家科研机构。全市工业用电量51亿千瓦时、增长14.5%，推进电力市场化交易，降低企业用电成本1.57亿元。

（二）加快推进乡村振兴，农业农村呈现新面貌

全面实施乡村振兴战略，大力发展农村经济，农林牧渔业增加值增长5.7%，创10年来新高。

现代农业稳步发展。以创建现代特色农业示范区为抓手，加快农业产业化、标准化建设。新建各级现代特色农业示范区157个，实现县（区）有示范区、镇有示范园、村有示范点，示范引领作用进一步增强。培育发展新型农业经营主体，新增自治区农业产业化龙头企业9家、市级龙头企业5家，畜禽现代生态养殖场认证率达83%。粮食生产稳定增长，划定粮食生产功能区47万亩，超额完成自治区下达的耕地和基本农田保护任务。开展“大棚房”问题专项清理整治，遏制农地非农化。全市行政村集体经济年收入均超过3万元。

海洋渔业加快发展。“南珠产业振兴计划”初战告捷，形势令人鼓舞，养殖面积达2.8万亩，插核超过1000万贝，产量超过1000斤。引进台湾、香港一批渔农合作项目，桂台（北海）农渔业合作双创园、香港喜记“蟹将军”工厂化养殖等项目顺利推进。深水抗风浪养殖（核心）示范区成为广西最大的网箱养殖基地，产量达4.4万吨。远洋捕捞产量突破2万吨、增长134％。北海市获批国家海洋经济发展示范区，银滩南部海域入选国家级海洋牧场示范区，“北海生蚝”获国家地理标志保护产品，“北海沙虫”通过国家农产品地理标志专家评审。

县域经济不断壮大。合浦县地区生产总值增长8.3%，财政收入增长17.7%，发展步伐加快。合浦工业园“一镇两园”、铁山东港产业园基础设施加快建设，海丝首港、嘉善现代产业园等一批项目落户合浦。铁山港区南珠新城完成规划、启动建设，南康特色小镇建设取得新进展。“宜居乡村”建设扎实推进，村级服务中心建设通过自治区验收，农村改厕改厨、危房改造工作走在全区前列。

（三）深入创建国家全域旅游示范区，旅游产业谱写新篇章

以创建国家全域旅游示范区为契机，加大旅游发展力度，推动旅游产业全面发展。加强旅游宣传推介，成功举办多次高规格、影响大的旅游营销活动，“面朝大海·心仪北海”旅游品牌效应不断扩大。投入1亿多元培育航线，现有国内航线29条、通达城市33个，北海机场旅客吞吐量达228万人次、增长33%，在全国229个民航机场的排位上升到第59位，跨入中型机场行列。新开通北海至北京、成都、大理的直达动车，北海火车站每天开行动车最多时达36对，全年旅客到发量999.3万人次。全面启动银滩“6+N”提升改造工程，加快建设高星级酒店群。抓紧编制涠洲岛“五规合一”总体规划，涠洲岛南湾海上运动基地入选广西体育旅游示范基地，涠洲岛帆船及游艇海钓线路入选广西体育旅游精品路线，在北海举办的全国翻波板锦标赛（总决赛）及全国OP帆船冠军赛入选广西体育旅游精品赛事。中信国安水乐园、希尔顿逸林酒店、冠岭旅游综合体等一批文旅项目建设进展顺利。签约引进银基、当红齐天等17个重大文旅项目，总投资1340亿元。加大资源整合和规划指导，乡村旅游加快发展。开展旅游市场整治“利剑”行动，游客满意度大幅提升。全年接待国内旅游者3935万人次、增长28.2%，国内旅游消费499.7亿元、增长37%，北海荣获“年度最受游客满意的中国旅游目的地”和“新时代·中国最佳文化旅游名城”称号，合浦县和涠洲岛旅游区成功创建广西特色旅游名县（区），鳄鱼山景区入选2018中国品牌旅游景区。

（四）积极发展新业态，高端服务业绘就新画卷

高端服务业初显成效。创新发展思路，谋划发展以金融业为龙头的高端服务业。制定《关于加快现代金融服务业开放发展的实施意见》，在全国率先出台简洁明了、操作性强的高端服务业优惠政策，规划设立6个高端服务业聚集区，引进金融科技类企业26家，京东云、敦煌网、新奥集团南方总部、百乘金科等50个高端服务业项目落户北海，石基信息第三方支付牌照上线运营。中电北部湾信息港、高新区软件与信息服务集聚区入驻企业264家，软件信息服务业总产值排全区第二位。

商贸及其他服务业平稳发展。促进批发零售住宿餐饮业加快发展，开工建设吾悦广场商业综合体、北海国际农商冷链产业小镇，建成3个市、县（区）级电商公共服务中心，村级电商服务站点实现全覆盖。金海湾红树林生态旅游区获批国家级服务业标准化试点。第三产业增加值增长11.7%，全市社会消费品零售总额增长7%，居民消费价格指数增长1.4%，金融机构本外币存款余额1113亿元、增长17.6%，本外币贷款余额778亿元、增长15.7%，金融存款增速稳居全区第一位。

（五）大力优化营商环境，招商引资实现新突破

招商引资成效突出。招商引资取得前所未有的骄人成绩，在全区一枝独秀。全年累计开展招商活动1015批次、对接企业2351家、接待客商1407批5254人次，新签项目271个、总投资3195亿元，引进重大项目个数居全区第一，其中百亿级项目10个、十亿级项目69个。在全区率先绘制电子信息、石油化工、临港新材料、海洋产业、林纸和木材加工、食品加工、高端服务业等7大“产业树”全景图，按图索骥精准招商，得到了自治区领导的高度肯定。

营商环境明显优化。全面开展优化营商环境大行动，严肃整顿工作作风，对优化营商环境不力的有关部门领导班子进行严肃处理，自治区党委主要领导在全区经济工作会议上给予点赞。对照营商环境14项考核指标，对标全国最高水平，优化审批流程，压缩审批环节和审批时限，审批效率大幅提升。推进“一事通办”改革，“最多跑一次”事项比例达98%，在全区率先实现一个工作日完成企业注册登记。投资项目“五环相扣、联审联批”模式获自治区党委主要领导肯定性批示，并向全区推广。

（六）创新办法解决难题，项目建设掀起新高潮

把项目建设作为工作的重中之重，千方百计打破用地难、融资难、审批难的瓶颈，有效推进项目建设，信义玻璃产业园等120个重点项目开工，惠科移动智能终端一期等85个项目竣工。科学谋划运营土地资源，激活房地产市场，创新征地搬迁安置补偿办法，最大限度让利于民，促进和谐搬迁、幸福安置。召开规委会18次、土审会12次，分别研究了259个和172个议题，解决了一批积压多年的项目规划建设问题，释放了项目建设潜能。全年收储土地3690亩，盘活存量建设用地15255亩，土地出让收入65亿元、增长396%，为项目落地、产业发展提供用地和资金保障。建立领导联系项目、部门并联审批、项目联审联批和“该干即办”等6项制度，成立项目帮办中心，选派百名干部服务百个项目，为项目建设提供全程化服务。

（七）突出抓好产业扶贫，脱贫攻坚取得新进展

投入财政专项扶贫资金3.11亿元，全面落实扶贫政策，按照“三员九有”要求，扎实推进各项工作。引进一批产业扶贫项目，实现对有发展能力的贫困户产业扶贫全覆盖。建设农村硬化道路256公里，完成贫困户危房改造189户。22个贫困村、1507户6722人达到脱贫摘帽标准，全面完成2018年脱贫攻坚任务。

（八）加强规划建设管理，城乡建设呈现新格局

以创建全国文明城市为抓手，深入推进“城乡环境提升年”活动，加大基础设施建设力度，提高城市管理水平，完善城市功能。推进北海市总体城市设计，完成多个重点片区控规和一批专项规划。贯通长沙路、七星江桥等12条路桥，开工建设西南大道东延线、江苏路等17个项目，改造小街小巷41条。市花公园、体育公园建成，中山公园、长青公园改造完工。审批建设中加广场、北海之星等10幢超高层建筑，已开工建设4幢。创新城市管理方法，实行精细化、网格化管理，数字城管指挥中心投入试运行。创立“北海e眼”综合执法指挥管理平台，让广大人民群众成为城市管理监督员。居民养犬专项治理工作成效显著，群众拍手叫好。城区机械化清扫率达到90%，环卫工作实现“三个广西第一”，城市环境卫生明显改善。开展交通秩序综合治理，交通乱象得到有效整治，文明礼让行为渐成风尚。

（九）改革再深入、开放再扩大，改革开放释放新活力

各领域改革持续推进。银滩旅游度假区管理体制、城区建设项目审批体制改革及国资国企改革稳步推进。新一轮市级财政体制改革、国有林场改革顺利完成。农村土地承包经营权确权登记颁证工作基本完成，在全区率先实现颁证率超90%。深化“多证合一”登记制度改革，推行证照分离改革试点，全市市场主体总量达13万户、增长28%。乡镇“四所合一”、公立医院改革加快推进，全市所有具有住院条件的基层定点医疗机构接入国家异地就医结算平台。出台《综合行政执法体制改革方案》。“政采云”平台在全区率先实现全市覆盖，“农事网通”“阳光红页”上榜2018年互联网治理创新十大优秀案例。

对外开放不断扩大。全口径实际利用外资到位资金6.9亿美元、增长39%，加工贸易进出口总额205亿元、增长54%。外贸进出口总额达315亿元、增长37%，创历史新高。铁山港作业区完成货物吞吐量2033万吨、增长8.5%，其中外贸进口货物吞吐量893.5万吨、增长34.6%，创历史最好水平。组织编制《北海市向海经济发展规划》，国家海洋四所正式运行，开展多项向海经济基础理论研究。积极参与“一带一路”和西部陆海新通道建设，主动融入“南向、北联、东融、西合”开放发展新格局，26个项目列入自治区“一带一路”西部陆海新通道项目计划。玉港合作园、桂台产业合作北海示范区加快建设。设计时速350公里的合浦至湛江高速铁路前期工作顺利推进。成功举办庆祝自治区成立60周年活动、第15届中国—东盟博览会中国“魅力之城”系列活动、第六届中国—东南亚国家海洋合作论坛、海上丝绸之路（北海）旅游产业发展投资大会等一系列重大活动，展示了北海开放合作新形象。

（十）扎实推进环境保护，生态建设厚植新优势

生态环境是最公平的资源，我们把环境保护作为最普惠的民生全力推进。开展污染防治攻坚战三年行动计划。认真落实中央环保督察“回头看”整改要求，采取68项措施整改6大类14个问题。铁山港码头堆放冶炼废渣问题全面整改到位，10多年来没能解决的牛尾岭水库饮用水水源地环境不达标问题得到有效解决，完成北郊水厂水质提标工程，实现地表水并网供水。组织实施《北海市涠洲岛生态环境保护条例》《北海市沿海沙滩保护条例》，划定海岸线300米控制线，在控制范围内临海一线未出让用地不得建设居住、工业仓储性质建筑。红坎污水处理厂和合浦县城、铁山港污水处理厂提标改造建成运行，全面完成自治区下达的镇级污水处理厂建设任务，清理整治41个直排口，消除了外沙渔港等3个黑臭水体，近岸海域水质保持优良。南流江流域环境综合整治项目加快实施，水环境质量达到自治区考核目标。市污水治理项目投入商业运营，市污泥集中处置中心主体工程基本完成，滨海国家湿地公园（冯家江流域）水环境治理工程开工建设。建立覆盖市、县、乡（镇）、村四级联动的河长制体系。全市空气质量三项重要指标均居全区第一位，空气质量优良天数343天，优良率为94%。

（十一）持续改善和保障民生，社会事业开拓新局面

累计完成民生支出130.7亿元，增长8.2%。城镇居民人均可支配收入增长6.4%，农村居民人均可支配收入增长9.8%。十件为民办实事30个子项目完成28个，另有2个因规划调整未能按进度完成。

教育事业加快发展。合浦县、海城区、银海区顺利通过义务教育均衡发展国家督导评估，进入广西教育第一方阵。深入开展“童子功1+N”素质教育，实施教育优秀人才“千人计划”、第三期学前教育行动计划和普及高中攻坚计划。市第十一中学、海城区第一实验小学等9所中小学、幼儿园建成使用，签约引进20所非盈利性高端品牌幼儿园，中法教育合作项目落户北海。规划建设15平方公里的职业教育园区，广西传媒职业学院签约入园。北海职业学院、桂电北海校区办学成效显著，招生人数和在校生规模快速增长。

医疗卫生服务不断改善。积极发展紧密型医联体，加强基层医疗卫生机构能力建设，推行药品采购“两票制”，群众县域内就诊率达90%以上，群众就医获得感持续提升。国家精神卫生综合管理试点工作经验在全区推广。制定《北海市名医工程实施办法》，建立“石学敏院士专家工作站”，北医康复医院、爱尔眼科医院等民营医疗机构投入使用，恒大养生谷、佰培恩医院等项目落户北海。持续深化生育服务管理改革，妇幼健康核心指标持续向好。

文化体育事业健康发展。组织开展各类群众文化活动和惠民演出3000多场，“戏曲进校园”覆盖率100%，“文艺精品月月演”场场爆满，《珍珠公主》《珠还合浦》入选自治区级文艺精品。海上丝绸之路·北海史迹申遗工作扎实推进，《碧海丝路》入选国家艺术基金传播交流推广项目，在北海取景拍摄的电视剧《沧海丝路》《北部湾人家》在央视播出，北海作家获第八届冰心散文奖。《唱响新时代·元宵特辑》北海元素推介提升了北海知名度、美誉度和影响力。成功举办环广西公路自行车世界巡回赛北海赛段赛事，我市运动员参加国际和全国性体育赛事获金牌18枚。市工人文化宫改扩建、老干部活动中心建成使用，市图书馆新馆、体育馆主体工程基本完成。规范整治网吧，全市423所中小学开展声势浩大的“拒绝网吧 拒绝网游”护苗专项行动。

创新能力不断增强。创建国家海洋经济创新发展示范城市取得明显成效，13个示范项目顺利推进。大力扶持中小型科技企业发展，全市企业孵化器面积超过21万平方米，入驻企业超600家。银河科技城被评为全区首批特色创新示范小镇，26家企业被认定为科技型中小企业。每万人发明专利拥有量5.6件。

社会保障持续完善。城市最低生活保障标准提高到每人每月620元，农村最低生活保障标准提高到每人每年4000元。城镇新增就业2.2万人，农村劳动力转移就业新增0.98万人，城镇登记失业率2.06%，企业职工养老保险参保人数增长15%，居民养老保险参保人数增长13%，居民医保财政补贴提高到每人每年490元，大病保险筹资标准提高到每人每年53元。棚户区住房改造开工建设1893套，基本建成498套，分配入住公共租赁住房2045套，入住率达95%，均超额完成目标任务。

社会治理深入推进。社会大局和谐稳定，人民群众安全感和满意度居全区前列。实施公安“六大工程”，着力提升“十大能力”，创新“党建+警务+N”社会治理新模式，扫黑除恶专项斗争取得良好进展，圆满完成一批重大安保任务，社会治安持续好转。采取“十个一律”的霹雳手段重拳打击传销，铲除了盘踞多年的传销顽疾，无传销小区覆盖率超过96%，全国、全区打击传销工作现场推进会在北海召开。加强法治宣传教育，市民法治意识不断提高。信访基础设施标准化规范化建设成效明显。落实民族区域自治制度，依法加强民族宗教事务管理。加强食品药品安全治理，广西食品安全城市创建工作顺利推进。建成市政府应急平台和航空应急救援基地。安全生产各项举措得到有效落实，安全生产形势持续稳定向好。

（十二）加快职能转变，政府建设树立新形象

坚持和加强党的全面领导，严格履行全面从严治党主体责任，进一步落实政治建设重点任务。深化“放管服”改革，整顿机关工作作风，不断提高政府效能。推进法治政府建设，坚持依法行政，重大行政决策机制进一步健全，行政监督和审计监督得到加强，仲裁改革创新取得新成效。深入推进政府系统廉政建设，坚决贯彻中央八项规定及实施细则精神，严格落实“三重一大”集体决策制度。认真执行市人大及其常委会的决议、决定，依法接受市人大及其常委会的法律监督、工作监督，自觉接受市政协的民主监督，人大代表建议、政协提案办结率和满意率均达100%。

一年来，外事、侨务、民政、地方志、档案、新闻出版、妇女儿童、残疾人、国防动员、海防管控、人民防空、优抚安置、双拥共建、防震减灾等工作取得新进展。

各位代表！过去一年，面对宏观经济下行压力持续加大的形势，我们积极应对、勇于担当，科学谋划崭新未来，抓住机遇、创造机遇，解决了不少难题，办成了许多大事，北海发展焕发出勃勃生机！奋斗凝聚艰辛，成绩来之不易。这是市委正确领导的结果，是市人大及其常委会和市政协监督支持的结果，是社会各界鼎力支持的结果，是全市广大干部群众团结奋斗的结果。在此，我谨代表市人民政府，向全市广大干部群众，向人大代表、政协委员，向各民主党派、各人民团体、各界人士和驻市单位、驻市部队，向所有关心支持北海建设发展的同志们、朋友们，表示衷心的感谢，致以崇高的敬意！

船到中流浪更急，人到半山路更陡。我们清醒地看到，北海仍处在爬坡过坎的阶段，发展中还有不少困难，工作中仍有许多不足。产业发展总体上处于中低端水平，工业存在短板，服务业水平不高，城市功能不够完善，部分重点项目建设推进不快，生态环境、医疗卫生、教育等民生建设与群众的期待还有差距，政府工作效能有待提高。我们将以问题为导向，以抓铁有痕、踏石留印的韧劲，认真加以解决。

二、2019年工作安排

当前，国际形势发生深刻变化，外部环境复杂严峻，不稳定不确定因素增多，国内经济形势稳中有变、变中有忧，面临下行压力。虽然我市去年招商引资成绩斐然，项目建设进展良好，但项目从落地到建成生效还有一个过程，更艰难的工作还等着我们，更艰巨的任务还在后头。我们必须求真务实、真抓实干、齐心协力，一个一个问题去解决，一个一个项目去推进，一件一件工作去落实，全力以赴推动北海高质量发展。

今年政府工作的总体要求是:以习近平新时代中国特色社会主义思想为指导，全面贯彻党的十九大和十九届二中、三中全会精神以及中央、全区经济工作会议精神，按照市委十一届七次全会的决策部署，深入落实“三大定位”新使命、“五个扎实”新要求和打造向海经济新任务，坚持稳中求进工作总基调，坚持新发展理念，坚持高质量发展要求，统筹推进稳增长、促改革、调结构、惠民生、防风险工作，进一步稳就业、稳金融、稳外贸、稳外资、稳投资、稳预期，打好三大攻坚战，推动产业高质量发展，深化市场化改革，扩大高水平开放，建美管好滨海城市，为全面建成小康社会收官打下决定性基础，以优异成绩庆祝中华人民共和国成立70周年。

今年全市经济社会发展的主要目标是:地区生产总值增长8%左右，财政收入增长7.5%左右，固定资产投资增长8%左右，规模以上工业增加值增长8%左右，外贸进出口总额增长9%左右，社会消费品零售总额增长8.5%左右，城镇居民、农村居民人均可支配收入增长8%左右，城镇新增就业人数1.6万人，城镇登记失业率控制在4%以下，居民消费价格指数控制在3%左右，全面完成自治区下达的能源消费总量、强度“双控”和控制温室气体排放任务。

我们将重点做好以下工作：

（一）着力做强优势特色产业，推动主导产业高质量发展

按照“强龙头、补链条、聚集群”思路，采取有效措施，推动电子信息、石油化工、临港新材料、海洋产业、林纸和木材加工、食品加工等产业发展壮大，挺直产业之“干”，伸展产业“枝叶”，促进产业转型升级、创新发展。

推动电子信息产业集聚发展。全力推动惠科电子北海产业新城项目建设，重点推进200亿元投资、2000亿元产值、6.8万人就业、200家配套企业的移动智能终端项目和智能电视机项目建设，打造全球最大的智能电视机生产基地和西南最大的移动智能终端生产基地。通过中电北部湾信息港引进100家以上企业，通过京东集团引进50到80家电商企业，加快发展软件和信息服务业。促进三诺、冠捷、石基、建兴等电子信息企业加快发展。推动北海威六科创中心在广西率先开展IPv6示范应用，加快建设IPv4-IPv6互联互通云中心、IPv6大数据应用与孵化平台，超前布局下一代互联网产业。

推动石化产业扩能升级。开工建设北海炼化结构调整项目，扩大北海炼化生产能力，优化产品结构，提高经济效益。按照“产业树”全景图开展精准招商，引进一批石化产业中下游项目，推进新鑫能源、LNG冷能综合利用等配套项目，建成北海至山口成品油管道项目，延长产业链。

推动临港新材料产业加速壮大。加快建设铁山港临港新材料产业园，引进一批下游不锈钢制品企业，规划建设剪切配送、交易、研发中心。继续推进诚德“填平补齐环保”项目建设，完善环保设施，促进企业绿色发展。加快建设信义玻璃产业园，推动信义玻璃项目（一期）年内建成投产，开工建设光伏玻璃项目。促进林纸和木材加工业提质增效，加快引进液体包装盒项目。

推动海洋产业蓬勃发展。全面实施《北海市向海经济发展规划》，以国家海洋经济发展示范区和海洋牧场示范区为载体，推动海洋产业转型升级，加快发展向海经济。提升发展海洋渔业，加强养殖规范管理，发展设施渔业，扩大生态养殖，稳步发展海洋捕捞。大力发展水产品精深加工，提高水产品附加值。全面振兴南珠产业，加快创建南珠标准化示范基地，建设白龙南珠特色小镇，推动南珠养殖、加工转型升级发展。加快推进国家海洋四所、北海海洋产业科技园区、桂台（北海）农渔业合作双创园等平台建设，启动银海区海产品环保加工产业园、北部湾渔业装备产业园等项目，培育海洋新兴产业。

推动园区经济加快发展。加强产业园区基础设施建设，完善公共服务，加快产城融合，不断提升承载能力。创新园区管理体制机制，鼓励发展飞地经济，提高园区自我发展能力。围绕龙头企业和重点产业开展招商，为企业和项目建设提供“保姆式”“顾问式”服务，推动产业集聚发展。力争北海工业园区升级为国家级经济技术开发区；推进北海高新技术开发区福成新区建设，争创国家自主创新示范区；完善北海综合保税区功能，实施新一轮加工贸易倍增计划；发挥铁山港（临海）工业区优势，发展壮大临港产业；加快推进铁山东港产业园、合浦县工业园及月饼小镇建设，打造工业强县发展平台。

（二）着力实施乡村振兴战略，推进农业农村加快发展

坚持农业农村优先发展，落实《北海市乡村振兴战略规划》和配套措施，打好打赢脱贫攻坚战，推动农业全面升级、农村全面进步、农民全面发展。

促进现代农业提质增效。深化农业供给侧结构性改革，提高农业产业化水平，稳定粮食生产，保障优质安全农产品供给。加强涉农项目招商，推动土地流转、土地托管、土地入股，扶持发展家庭农场、农民合作社等新型经营主体，发展多种形式的适度规模经营。促进现代特色农业示范区增点扩面提质升级，打造北海生蚝、沙虫、海鸭蛋等农产品品牌。加强水利基础设施建设，加快推进总江水闸、旺盛江水库、南流江治理河势控制工程、海湾新城乾江围、合浦县百曲围、银海区海陆海堤等项目建设。

扎实推进脱贫攻坚。按照“两不愁、三保障”的总要求，进一步巩固成果、提升质量，高水平完成自治区下达的脱贫攻坚任务。加强“一帮一联”工作，坚持扶贫同扶志、扶智相结合，激发贫困群众内生动力。落实农村最低生活保障、贫困失能人员集中供养等兜底政策。统筹使用各级各类扶贫资金，抓好贫困村道路硬化、危房改造、人饮安全等建设，加快发展特色产业，村级集体经济年收入超过4万元。切实把国家、自治区历次巡视、督查、评估、考核反馈的问题整改到位，严肃整治扶贫领域腐败和作风问题。

统筹推进城乡一体化发展。统筹谋划县区发展，支持县区改善发展条件，推动县区工业和特色产业发展壮大，加快传统产业转型发展，促进县域经济做大做强，推进城乡一体化发展。加快建设豹狸村“龙窑古镇”田园综合体，打造乡村振兴示范点。实施乡村风貌提升三年行动，开展“幸福乡村”活动，加快编制实用性村庄规划，建立乡村规划建设管理长效机制，因地制宜发展特色产业，着力打造“生态文明建设示范村镇”，改善群众生活生产环境。

（三）着力创建国家全域旅游示范区，促进旅游产业提档升级

以创建国家全域旅游示范区为抓手，推动景点旅游向全域旅游转变，实现旅游业全域共建、全域共融、全域共享。

突出抓好旅游项目建设。加快推进银基、当红齐天、海丝首港等重大文旅项目落地建设。高标准推进银滩提升改造，加快建设银滩“6+N”项目和一批休闲街区、主题游乐项目，擦亮北海滨海旅游金字招牌。完善涠洲岛“多规合一”，高水平推进保护开发。加快建设北部湾邮轮母港，打造海上丝绸之路旅游品牌。推动金海湾红树林生态旅游区、园博园等景区提档升级，创建一批高等级景区、乡村旅游区和旅游特色小镇。

创新发展“旅游+”模式。适应传统旅游向现代旅游转型的趋势，通过发展“旅游+”，创新和丰富旅游产品，催生旅游新业态，抢占未来旅游发展制高点。加快发展旅游+文化，打造名人小镇，举办高端文化艺术交流活动，发展名人经济。发展旅游+体育，推进体育冬训基地建设、举办国际性大型体育赛事，推动旅游和体育融合发展。发展旅游+医疗康养，加快推进恒大养生谷、万润高端医疗综合体等项目建设。发展旅游+农业，加快建设田园综合体，促进乡村旅游发展。加快开发滨海休闲、海岛度假、海洋体验、海丝文化、避寒养生等特色休闲度假旅游产品，满足旅游消费转型升级多样化需求，形成北海旅游新亮点，打造滨海特色旅游品牌。

加快提升旅游服务水平。与国内著名旅游信息化公司合作，建设全域旅游大数据中心和旅游市场监管服务平台，推进旅游服务和管理智能化、信息化。加强与国内外知名旅行社合作，拓展客源市场，提高旅游业经营水平。加强特色旅游商品的设计、生产，对旅游商品经营实行标准化管理。整治旅游市场突出问题，规范旅游市场秩序。加强与春秋航空等航空公司合作，继续加密航班、开拓航线，扶持发展水上飞机、通用航空等新型航空业态，力争开通直达深圳等城市的动车，改善游客通达条件。

（四）着力发展高端服务业，培育经济发展增长点

高端服务业是城市发展质量和综合竞争力的集中体现，也是吸引人才、留住人才以及提升城市未来竞争力的重要载体。我们要着眼北海长远发展，立足北海城市特质、产业基础和市场潜力，创新发展以金融业为龙头的高端服务业，打造支撑北海赶超跨越和高质量发展的主导产业。

促进高端服务业加快发展。重点培育发展总部经济、金融服务、生产性服务、软件和信息技术服务、科技服务、现代物流、商务服务、文化创意8个领域的高端服务业。加快建成6个高端服务业集聚区，全面落实优惠政策，引进一批高端服务业项目，加快推进京东云大数据中心等项目建设，促进信义集团物流中心、结算中心与供应链管理、国际贸易业务落户。扶持发展现代金融服务业，加快推进北海市红树林金融科技小镇建设，引进一批金融类企业，以新一代电子信息技术、大数据、人工智能、区块链为支撑，充分发挥科技和金融资源的集聚优势，提升金融服务科技化、专业化、产业化水平，规划建设北海金融商务区，打造现代金融活跃示范区。

促进商贸服务业升级发展。改善消费环境，鼓励消费升级，促进消费增长。出台鼓励措施，鼓励商业综合体、商品交易市场、物流企业加快发展，规划建设大型批发市场，激发批发零售住宿餐饮行业发展活力。推动北海国际农商冷链产业小镇建设，打造广西规模最大的农商冷链物流产业集群。积极发展电子商务，推动电商进企业、进农村，拓宽北海特色商品销售渠道。

（五）着力发展民营经济，增强发展内生活力

民营经济是国民经济的重要组成部分，是我市创造税收、吸纳就业、促进发展的重要力量。要以更加开放的姿态吸引民营资本、拥抱民营企业，毫不动摇鼓励、支持和引导民营经济发展。加大对企业的扶持力度，落实减税降费政策，降低企业要素成本，切实减轻企业税费负担。建立完善中小微企业融资担保体系，落实“4321”风险分担及代偿补偿机制，发挥产业基金作用，对有发展潜力的项目进行风险投资，缓解民营企业特别是中小微企业融资难、融资贵问题。畅通政府与民营企业沟通渠道，营造公平竞争环境，尊重、关心、支持企业家干事创业，构建“亲”“清”新型政商关系，让更多优秀民营企业家脱颖而出、健康成长，推动民营经济发展壮大。推动大众创业、万众创新，发挥企业创新主体作用，聚集创新资源，加强产学研合作，加快科技创新，促进民营企业创新发展。

（六）着力抓好精准招商，积蓄经济发展动能

招商引资是推动北海发展的命运工程。要大力优化营商环境，按照7大“产业树”开展精准招商、重点招商，不断提高招商引资成效。

提高精准招商成效。重点围绕引进工业和高端服务业项目开展招商，赴上海、广州、深圳、香港等地开展大型主题招商活动。创新招商引资方式，完善考核激励机制，激发园区、县区、部门招商积极性。发挥龙头企业、产业平台以及行业协会的作用和优势，开展以商招商、渠道招商及点对点招商。进一步加强招商服务，确保招商项目能够引进来、落得下、留得住、发展好。

持续优化营商环境。开展“优化营商环境突破年”活动，推进“一事通办”改革，对标全国先进，精简审批事项，再造审批流程，提高审批效率。推进互联网+政务、+监管建设，加快打造数字政务一体化平台，建好“一张网”，只进“一扇门”，实现“最多跑一次”。加快社会信用体系建设。聚焦企业和群众关注的热点、难点问题，深入开展政务服务、公共服务窗口制度化、规范化、标准化建设，提高企业和群众满意度，努力打造一流营商环境。

培育企业生长环境。企业生长环境是营商环境的升级版。积极培育发展资本、技术、人才三大要素市场，打造良好的企业生长环境，这是北海超前布局、增强城市未来竞争力的战略性举措。加快培育资本要素市场，通过建设北海市红树林金融科技小镇，打造便于科技型公司资本筹集、融资聚集的平台；成立产业发展基金，撬动社会资本，放大资金效应，让资本助力企业加快发展。加快培育技术要素市场，通过与国家技术转移中心合作，建设北部湾国际技术成果转化中心；联合国内著名高校、科研院所及知名企业，举办国际技术转移博览会，打造高端技术交易市场。加快培育人才要素市场，通过建立高端人才网，鼓励支持企业引进高层次人才、创新领军人才，打造科研人才的集聚地；实施蓝海战略，大力发展职业教育，加快建设职业教育园区，引进社会资本建设海洋职业学院、电子实用技术职业学院、旅游职业学院等10所职业学院，推动北海职业学院和北海市卫生学校升级，加快形成“10+2”职业教育集群，为企业提供实用型、熟练型技术人才。同时，大力引进物流龙头企业，完善物流配送体系，发展智慧物流，降低企业物流成本，建设便捷高效和低廉的物流环境。

（七）着力推动项目建设，夯实经济发展后劲

组织实施“项目落实年”活动，落实重点项目动态管理制度，围绕项目落地和建设，持续发力、集中攻坚，促进在谈的尽快谈成、签约的尽快落地、开工的尽快投产，实现项目建设快落实、大突破。突出抓好征地搬迁，抽调精兵强将组建18个征地工作组，实行“一个项目、一名领导、一支队伍、一抓到底”的“四个一”机制，倒排工期、挂图作战，全力以赴保障项目用地。科学运营土地，稳定土地供应市场，筹措项目建设资金，完善项目配套基础设施。加快推进130多个重点项目开工建设，加强服务信义玻璃产业园、中电北部湾信息港等一批在建项目，不断掀起项目建设高潮。

（八）着力加快城市建设，优化城市功能品质

扎实推进全国文明城市创建工作，着力完善城市功能，提升城市精细化管理水平，建美管好滨海城市。

拓展城市空间布局。加强规划管理和城市设计，启动国土空间规划编制，按照“一岛、两带、三港、四路、五组团”的布局，拉开城市框架。开工建设高德至大风江滨海大道，将主城区往合浦方向延伸；改造提升向海大道（北铁公路），加快把主城区与铁山港区连为一体；开工建设西村港大桥，加快把银滩东区与海洋产业科技园区连成一片。启动冯家江区域和廉州湾区域两个城市CBD建设，加快建设10幢超高层建筑，推进银滩新城、廉州湾新城、海洋新城、南珠新城等重要片区建设，促进产城融合发展。

完善城市基础设施。持续推进城市畅通工程，加快建设西南大道东延线等市政道路，贯通和完善城市路网。推进人行道净化以及小街小巷改造，打造严管街、示范路。启动城市夜景亮化工程，推进城市绿化、彩化、美化。积极发展公共交通，加强公交站点建设，合理调整公交线网。建设一批公共厕所、小公园、小绿地、小广场。推进黄金北岸、南珠市民休闲广场、冠岭公园改造和高德老街改造等项目建设。推动合浦至湛江高速铁路、北海机场停机坪扩建等基础设施建设，加快铁山港集装箱码头和公共执法码头建设。规划建设智慧城市，加强新一代信息通信基础设施建设，推动城市智能化、高效率和可持续发展。推进海绵城市建设，完善城市内涝防治体系，加快地下综合管廊建设。加快城市生活污水处理设施建设与改造，推进产业园区工业污水集中处理设施建设，多点推进城镇污水管网补短板。推进生活垃圾焚烧发电和餐厨垃圾处理项目。完善电网规划，改造提升电网基础设施，加快推进涠洲岛跨海联网工程建设。

提升城市管理水平。推行城市综合行政执法，完善综合执法指挥监督平台，加强城市网格化、精细化管理，提升城市管理信息化、智能化水平。发挥“北海e眼”作用，调动群众参与城市管理积极性和主动性，落实“门前三包”制度，推动城市基层管理由街道模式向社区模式转变，形成城市管理合力。规范住宅小区管理，加强业主依法自我管理与和谐社区建设。

（九）着力加强生态建设，提高生态宜居水平

绿色生态是北海最突出的优势、最宝贵的财富、最有效的“王牌”。牢固树立“绿水青山就是金山银山”理念，深入开展蓝天保卫战、清水行动和净土行动，守住生态红线，保持生态优势。

严格落实生态环境保护“党政同责、一岗双责”责任制，优先安排资金保障生态建设，深入实施污染防治攻坚战三年行动，着力提升大气、水、土壤等污染的治理水平。巩固提升中央环保督察“回头看”反馈问题的整改成效，严肃处理发生在群众身边的、群众反映强烈的污染问题。建立完善污染监测预警体系，加强县区环境监测能力建设。加大力度对自然保护区、近岸海域、入海河流、采砂采矿等方面巡查监管，治理南流江、牛尾岭水库、旺盛江水库、洪潮江水库等重点河湖环境，推进饮用水水源地及第二水源工程建设，确保饮用水供给安全。大力整治银滩核心区污水管网，推进冯家江湿地公园、廉州湾海域清淤和生态岸线修复等综合整治工程。加快铁山港固废堆场项目建设。发展绿色产业和循环经济，引进节能环保、新能源应用和可再生能源开发企业入驻园区，推动发展环保服务业。

（十）着力深化改革扩大开放，增添城市发展动力

改革开放是北海实现高质量发展的动力源泉。要坚持改革不歇力、开放不停步，深化市场化改革释放新活力，扩大高水平开放增添新动能。

推动各领域改革。全面完成政府机构改革任务，持续深化“放管服”改革。继续推进市属平台公司改革，落实《北海市涠洲岛旅游区综合改革方案》，推进白沙镇经济发达镇行政体制改革试点工作。推进“证照分离”改革，深化财税金融改革，规范财政事权和支出责任划分。完成农村土地确权登记颁证和农村集体产权制度改革试点。深化医药卫生体制改革，推进市公立医院薪酬制度改革。统筹推进市场准入、社会管理、文化教育和社会保障等领域改革。

推动高水平对外开放。积极参与“一带一路”和西部陆海新通道建设，主动融入“南向、北联、东融、西合”开放发展新格局。完善口岸设施，加快“单一窗口”建设，提升贸易便利化水平。鼓励外资企业发展壮大，扩大利用外资规模。积极对接粤港澳大湾区、海南自贸试验区和环北部湾城市群建设，加强旅游、高新技术和现代服务业等方面的产业合作，促进北海开放发展。加强与周边城市的合作，融入区域一体化发展。加快推进两岸冷链物流产业合作城市、桂台产业合作北海示范区和玉港合作园建设。推进质量强市、品牌亮市建设，加强与“中国质量万里行”合作，策划推出一批名优特产，打造北海知名品牌。集中开展对外宣传推介活动，重点宣传展示北海良好的营商环境、生态宜居环境。主动承办国际国内高端论坛和大型企业年会，办好北海南珠节暨北海国际珍珠展，不断提升北海美誉度和知名度。

（十一）着力发展基础教育和医疗卫生事业，增强公共服务能力

优质的基础教育和医疗卫生，既是最大的民生需求，更是吸引投资、留住人才的关键一招，功在当下、利在长远。要推动教育卫生事业高质量发展，让群众共享公平有质量的教育和高水平的医疗卫生服务，吸引更多的优秀人才和投资者。

加快提升基础教育质量。巩固义务教育均衡发展成果，全面提升中小学幼儿园办学水平和质量。突出抓好德育和特色教育，与北京师范大学合作编写具有北海特色的德育教材，培养学生优良的品德、坚强的意志和强烈的责任心；继续在幼儿园、小学实施“童子功1+N”计划，在初中开设特色班，在高中集中优质资源，有针对性地培养特长生。开展基础教育质量提升行动，今年投入10亿元、改扩建10所中小学校；实施“三名”工程，引进一批国内名校到北海办学，加快提升名校长管理能力，引进和培养一批名教师、名班主任；实施教育优秀人才“千人计划”，强化师德师风建设，提升教师队伍综合素质。优先开展教育暖心工程，切实解决一批涉及学校供热、供电、供水、教室白板、师生出行的困难，协调一批急需解决的事项，提高乡村教师生活补助标准。

加快提升医疗卫生水平。大力实施“名医工程”，实行引进与自主培养相结合，柔性引进一批医疗卫生专业的顶尖人才，全职引进一批高层次、急需紧缺专业人才，培养一批专业后备人才，评选一批“北海名医”。建设院士专家工作站和名医专家工作室，凝聚一批高层次领军人才。重点围绕心脑血管疾病科、肿瘤科、妇产科、儿科、骨科开展工作，逐步拓展到其他专业学科。建设县域医共体、紧密型医联体，加强家庭医生签约服务，提升远程医疗服务水平。加强基层医疗卫生机构服务能力建设，推进乡村医生“乡聘村用”。加快建设涠洲岛医院，推进北海市人民医院异地扩建二期工程和海上紧急医学救援中心项目。落实两孩政策，保障妇幼健康。积极创建“全国卫生城市”，全面提升城市卫生管理水平。

（十二）着力繁荣社会事业，不断增进人民福祉

坚持权为民所用、利为民所谋，大力加强保障和改善民生，加大投入，补足短板，进一步提升群众获得感、安全感和幸福感。

大力发展先进文化。培育和弘扬社会主义核心价值观，加强公民道德建设，提升市民文明素质和城市文明程度。鼓励开展文艺创作，推出更多艺术精品，让文艺作品“接北海地气”“有北海底气”“为北海聚人气”。加强非物质文化遗产保护传承，加大投入保护利用好海上丝绸之路文化遗产，推进海上丝绸之路·北海史迹申遗。加强文化惠民，鼓励发展北海特色文化，丰富群众文化生活。加快发展文化产业和创意产业，发展传统工艺美术产业，推动海上丝路北海工艺美术博览园改造提质。培育艺术品交易市场，引进一批文化艺术名人。大力发展体育事业，广泛开展全民健身活动，积极举办国际性、全国性体育赛事，承办好“一带一路”国际帆船赛和环广西公路自行车世界巡回赛北海赛段比赛等，积极备战区运会、全国青运会。加快完善文化基础设施，建成市体育馆并投入使用，推进市博物馆、市档案馆、市委党校二期、市青少年宫和妇女儿童活动中心等项目建设，启动北部湾体育中心和市群艺馆搬迁项目。继续抓好“拒绝网吧 拒绝网游”护苗专项行动和扫黄打非五个专项行动。

努力提升社会保障水平。实施就业优先战略和更加积极的就业政策，抓好高校毕业生、农民工等群体就业工作，帮扶失业人员重新就业。做好退役军人工作，加快创建全国双拥模范城，积极推动军民融合发展。实施全民参保计划，扩大参保范围，实现应保尽保。扩大跨省异地就医联网结算范围，继续推行职工医保个人账户家庭成员共享。统筹城乡社会救助体系，落实最低生活保障制度。搭建智慧养老服务平台，提升养老服务信息化水平。加强保障性住房和公租房建设管理，让住房真困难的群众得到帮助。

切实加强和创新社会治理。落实法治建设任务，开展普法宣传教育，加强法治文化建设，增强群众法治意识，提高基层自治能力，构筑共建共治共享社会治理新格局。继续推进公安“六大工程”，提升“十大能力”；依法打击和惩治各类违法犯罪行为，加大“扫黑除恶”工作力度，严惩“保护伞”；保持高压态势打击传销，巩固打击传销成果，彻底铲除传销土壤，加快创建“无传销城市”，营造广西一流的社会治安环境。加强应急管理，完善灾害事故预防预警和应急处置体系。落实安全生产责任制，加强安全风险分级管控和隐患排查治理，遏制重特大安全生产事故发生。加强食品药品安全监管，全面创建广西食品安全示范城市。切实做好信访和调解工作，积极预防和化解矛盾纠纷。

各位代表！习近平总书记提出了以人民为中心的发展思想，民之所盼就是政府所想，民之所求就是政府所为，人民对美好生活的向往就是我们的奋斗目标，政府的一切工作都应该围绕人民、服务人民、造福人民。今年，我们将把75%以上的财力投入到民生领域，用于保障和改善民生，重点在生态保护、市政设施、社会保障、医疗卫生、基础教育、文化体育等方面建设一大批惠民项目，把人民群众最烦心的事情一件一件解决，把人民群众最关切的事情一件一件办好。

三、进一步加强政府自身建设

今年是北海发展的关键之年，我们要在新时代有新作为，必须践行“担当为要、实干为本、发展为重、奋斗为荣”的为政理念，建设法治、高效、廉洁政府，对人民负责、让人民满意。

坚持依法行政，努力建设法治政府。依法行使政府权力，落实“三重一大”集体决策制度，提高政府决策科学化民主化法治化水平。依法接受市人大及其常委会的法律监督和工作监督，执行市人大及其常委会的决议、决定，向市人大常委会报告工作、向市政协通报情况，配合市人大推进重点领域立法工作，自觉接受市政协的民主监督，支持政协履行职能，认真办理人大代表建议和政协提案。加强行政监督和审计监督，进一步完善仲裁、公证、法律顾问等公共法律服务体系。

坚持“该干即办”，努力建设高效政府。践行“三坚持两改变”，严肃整顿工作作风，整治置若罔闻、庸碌无为、冷漠懈怠、沾沾自喜、居功自满等行为，建立激励容错机制，强化督查督办，严肃考核问责，切实提高机关工作质量和工作效率，以锐意进取、奋发有为的精神状态，以勇于担当、敢于作为的使命感，以慢不得、等不起、坐不住的紧迫感，高标准推进工作，高质量完成任务。

坚持从严治党，努力建设廉洁政府。严格落实全面从严治党主体责任，树牢“四个意识”，坚定“四个自信”，坚决做到“两个维护”。持之以恒落实中央八项规定及实施细则精神，规范公共财政支出，坚持勤俭节约，少花钱多办事。加强政府廉政工作，严惩发生在群众身边的腐败问题，大力整治形式主义、官僚主义，以廉洁政府形象取信于民。

各位代表！伟大梦想不是等得来、喊得来的，而是拼出来、干出来的。让我们更加紧密地团结在以习近平同志为核心的党中央周围，团结拼搏，开拓奋进，担当作为，善作善成，加快建设富强开放生态文化幸福新北海，为建设壮美广西共圆复兴梦想作出积极贡献，以优异成绩向中华人民共和国成立70周年献礼！
